# Minutes of the Twenty-Third Annual Session of the Southern Dental Association

**Published:** 1892-02

**Authors:** 


AMERICAN JOURNAL

—OF—

### DENTAL SCIENCE.

Vol. xxv. Baltimore, February, 1892. No. 10.

ARTICLE I.

### MINUTES OF THE TWENTY-THIRD ANNUAL SESSION OF THE SOUTHERN DENTAL ASSOCIATION.

[Continued from page 404.]

HELD IN THE NORTH CAROLINA TEACHERS' ASSEMBLY HALL AT MOREHEAD, N. C., AUG. 11, AT 10.30 A. M.

AFTERNOON SESSION, AUGUST 11th, 1891.

The meeting convened at 4 p. m., President Wright in the chair.

The Committee on Operative Dentistry being called the following paper was read by the chairman.

### OPERATIVE DENTISTSY.

BY DR. E. L. HUNTER, ENFIELD, N. C.

As I address myself to the young or inexperienced in operative dentistry, I ask to be pardoned for a practical talk not intended for old and cultured thinkers and workers. If I am candid or dogmatic, or worry you with old or well-worn subjects, I beg, Mr. President, you will believe I am actuated by a sense of duty in the presence of what seems to be a need. If I hold before you and press on you those things which having passed the stage of theoretical and practical, are remembered only as A. B. C. Patiently remember that there *are* those who have forgotten, never know or, remembering, will not or do not.

The grand ideas of operative dentistry are the prevention and arrest of decay in human teeth. As a preventive it was thought that "bone-making material," such as the outer husk of wheat, rye and oats, would do something for us. Some years ago both medical and dental journals were full of literature on this subject. In vain science sifted and analized for, practically, the bone-making material was in the bone itself, *i. e.* in the individual—his vitality and power of assimilation. We have "become as gods, knowing good and evil," but we have ignored nature's laws of breeding ("the survival of the fittest") and taken for ourselves a system which we are unwilling to accord to our dogs, cows and horses. In many other ways we have, for ages, lived unnatural lives, so that when as in the best therapeutics we would remove "the cause" we turn helplessly from the contemplation and feebly address ourselves to results. It is true that great good may be done by inducing better systemic conditions through a better educated profession and laity, and in that respect it is probable the future holds some better things for us.

At present the duty of saving an endless number of decayed teeth looks us squarely in the face. To accomplish that duty is incumbent not only for the sake of the comfort, cleanliness, health and beauty of the individual, but to answer the ends of that broader humanity affecting our posterity. What will do this? Is it filling—bone filling, with any one of the various materials? Not at all, but yet filling, with a fair conception or the thereputic conditions past, present and future, adding thereto such mechanical art as will answer the ends sought. We may not be able to do away with the causes of decay, but we may remove the occasion *by leaving indestructible points of contact.* This contains a vital principle in tooth saving second to none. I know it is old, but it is not therefore less important or valuable. It makes no difference what material the circumstances have induced you to use,this idea should be strictly adhered to. What then would be the visible aspect of cavities prepared and filled in this way? Evidently margins so far away are the receding lines of the teeth as to be entirely free from contact with each other. To accomplish this it is frequently necessary to carry the cervical margin under the gum (or free margin) but I feel compelled to object to that as a rule. One, in that particular, should be governed a great deal by the shape and texture of the teeth. There is no doubt that in a great many cases the removal of tooth substance on approximate surfaces is insufficient for tooth preservation even where cautious work is contemplated, or else removing too much approximate material in finishing fillings, thus leaving rather flattened outlines and consequent approach of margins.

It does seem that in faulty nutrition as with dyspeptics and a majority of child-bearers, no amount of skill can always prevent a recurrence of decay In such cases I have observed good effects from both tin and amalgam, used to cover the cervical wall. I have kept no record but have used it many times and never had occasion to remove it once, either from stain or recurrence of decay. Theories are easy to advance and at times as easy in deluding, but practical facts are stubborn things. I have seen and made a great many gold and amalgam fillings (in the same tooth) and have never yet seen a failure where they were joined together. In fact the good has been so palpable that I have been unable to avoid according to it excellence. Soft teeth seem also to have played an important part in failures to arrest decay. A great deal has been said about soft teeth. In the long ago when Arthur spaces were made, when all the teeth were cut away on their bearing surfaces, and allowed to fall together on their thinly enameled necks, it was soft teeth. When the dentist and the people had grown tired of the file, the chipped corners and rotten crevix—when there was no cutting, but wedging for space, filling small crevices and allowing margins in contact, it was soft teeth. When they wedged some and cut some and made a labial crescent cut (to show some gold and look pretty) sand stripped away all the covering nature ever intended, allowing subsequent cervical, cutting edge and lingual marginal contact, and named it "contour work"—it was soft teeth. Indeed, gentlemen, soft teeth have been such a blessing to the dentist and such a curse to the people I don't know what we would do without soft teeth, anyhow. Almost every dentist would be skillful and the people blessed.

It seems late in the history of dentistry to speak of Arthur spaces, yet it is quite often we see where the disk bicuspid and molar files have recently been. These spaces so conveniently and cheaply made are destructive not only to opposing angular unprotected enamel corners and impinging cervical borders, but also result in somewhat contracted arches, especially when made in young subjects.

This lessening of the arch means the laying of all the flat cut surfaces over against each other with obvious results. Fortunately this custom is becoming, even in darker corners, rather unfashionable—the people are afraid of a man who wants to file their teeth. But there yet prevails, to a great extent, a practice offering the delusion of show and name, which, if it offers but little opportunity for contraction of the arch, is sufficiently destructive in subsequent decay to damn it, and that is *partial* contour work. I mean by partial contour work that which does not, by proper size and fullness prevent marginal contact. This, sometimes, results where there has been an honest effort to do proper contour work by reason of an attempt at quick and consequent insufficient separation, leaving not enough space after the filling had been built to finish with swell enough to prevent marginal contact. The effort to acquire and hold a large practice, the effort at quantity rather than quality, indeed, quantity at the expense of excellence is one of the sins for which we must answer. Nowhere, perhaps, is a competent dentist so completely handicapped as when working without abundant space. Let anyone who has been working with the space obtainable by separators, try slow wedging and get more space and see the difference, not only in the formation of cavity and introduction and condensation of material, but in the shape and finish of work. I do not wish to condemn separators altogether—in young subjects where the crowns are wide compared with the necks and the teeth move easily, quite sufficient space may be gained at the time with separators, and the filling done at once. I mean to condemn no good thing but to recommend that which experience has taught me to be inseparable from excellence in approximate work, *i. e.*, Space! Space to see, in which to exercise skill in filling, and space to finish with. Space is *the* condition under which alone skill *may* be exercised. Without it, skill is turned into the most absolute bungling. It is a fact that slow wedging requires time and time may lose us a patient as it often happens that there is no time and space, and the separator will not get it, it then becomes the question whether we will commit the sin or let some brother do it—we are too noble, too generous, we do it ourselves. I am aware that this is probing and picking the professional skin, but it is no more than fair the truth should be told sometimes.

I wish I could impress upon all young men the importance of subordinating all things to excellence, not only because it is right, but provident, for good principle and good policy are inseparable. Excellence in anything will in time work out all the attending good.

There is hardly any instrument, system or principle not open to abuse. Observe, one of the principle advantages in wide separation is that one may see how and have room to make good adaptation to cavity walls, particularly margins, and then adjust a matrix to make good wall adaptation extremely difficult and the margins of a properly shaped cavity, impossible. For amalgam the matrix is excellent, permitting better condensation in a shorter time, but it does not do away with the need to shape the filling and trim the margins afterward.

It was the near approach to artistic mechanical perfection which proved the truth of the underlying principle in contour work, since without it contour work is not good, and with it no other system is good. So art became the test of science. Only by a cultivation of art in conservative operative dentistry may we hope to establish principles. Indeed, a vast number of our principles never culminate in good except when applied with the most exquisite art. As an instance: Take nerve cappings and some root fillings. It is true there must be the precedence of discrimination between health and disease, and a knowledge of the thereputics, and yet with these things equally known to two operators, what is possible to one is entirely impossible to the other. I feel proud that American dentists have long been famous for their art, and prouder that art is still ascending; but it is a greater pleasure to think that science and art are both concomitants of American dentistry.

We have an endless number of principle, methods, materials and instruments, but our greatest need seems as much their *conservative* as skillful application. This is nowhere more apparent than in bridge work. Our tendency to run away with a good thing, to proclaim "a king cure all," to be fashionable to follow a fad—not to discriminate, has been our bane. It did seem to us that like plate work B. W. was not an unmixed good, and that it was to become needed only when the least of the evils; but the tendency has been not that, but to apply it wherever it was possible to do so, thus violating natural laws as well as the soundest principles of dental science. It is true enough that the stomach, intestines, kidneys, liver and even roots of teeth may be abused *some*, but is it not yet we have learned there must be a limit to the time and amount and a source? O, tempora! O, mores! When we had no choice, when it was a partial plate or nothing, we had to do what we could do. Now B. W. ought at least to offer us a choice between two evils. Are we not to discriminate?

### DISCUSSION.

Dr. Johnston, of Virginia, declining to open the discussion, Dr. Crawford responded, as follows:

Teeth do not only decay earlier in life but they continue to decay later in life than originally, in proportion as physiological conditions change the agents we use in the diseases peculiar to the teeth lose their valve. The point acted upon by the profession is the point of thoroughness in our operations, not in reference to one individual cavity, but in reference to the removal of diseased tissues from all cavities. If there is disease, it would be well to suggest, that if the dental tissues become involved, it ought to be corrected as early as possible. Very many of the cases of cures in the mouth of our patients are attributed to the presence of disease in some other portion of the system. Go to work and fill a few cavities nicely and call your friends in to look at them, they will speak of the margin being so smooth; they will speak of artistic beauty. Filling the teeth with gold, protecting the tissue and restoring the contour of the tooth as it was, is an artistic feat, and the skill that is necessary to bring about this is a high type of workmanship. To do this and still maintain a high therapeutic condition is a still more exalted step. The great trouble has been to harmonize this therapeutic and mechanical effect. My idea is contained in the paper just read, in regard to lining the walls with tin. Dr. Freeman, of Nashville, has attained a great degree of skill in the application of tin to the wall, but it is still more encouraging to see the results. That is the suggestion I have been acting upon for a good many years. The therapeutic effect of tin is very fine as a preservative.

There is one material referred to that I must raise my voice against, under a solemn sense of duty, but with profound respect to my brethren, and that is the use of amalgam. Its effects are next to the nefarious results of rubber, mark what I tell you. The science of dentistry has got to "about face" on the subject of amalgam, it will not do.

Seven years ago I stood before an intelligent audience and said that modern crown and bridge work was the coming practice, and thanked God that I have lived to see the demonstration, that modern crown and bridge work is one of the most successful practices of the day. We must practice dentistry in accordance with the better interests of patients and not for the shekels; make those who are able to pay, pay well, and those who are not able to pay, give it to them. A dentist wants to hear well; we want to see well and want to feel well. There is no class of people on earth whose sense of touch ought to be so exquisite as the dentists.

I hope this Association will take on a degree of enthusiasm and do better work than ever before. I hope crown and bridge work will have more consideration. There was one little suggestion I did not accede to, and that was that we might go too far in that respect. I don't believe there is a man on the face of this earth who has fully comprehended the efficiency of crown and bridge work. Some of us have given attention to it and developed it somewhat, but the heighth and depth of prosthetic dentistry has never been reached. I once heard of a case in which a bridge of fourteen teeth was constructed, with three natural teeth attachments, and for want of a fourth one, a tooth was implanted. The operator ran off on the idea that it was a wonderful thing! Can you conceive of a poorer condition of things, than a bridge of fourteen teeth, with three permanent attachments and the fourth one made by implantation? That would be one of the most successful cases of implantation ever known; but it is morally wrong and unscientific in character. God Almighty never conceived the idea of robbing Peter to pay Paul, and it will not be a success!

Dr. Hunter: I think I should be counted unworthy of trust if I did not measure swords with Dr. Crawford, especially when he says that amalgam is a great curse to the country. I happen to know that facts are stubborn things, by personal observation for a number of years, not confined to to-day, or yesterday or twenty years, and amalgam has done good service. I can't say I can make amalgam fillings in some cases, but discrimination is a great thing, and it is a great comfort to thousands of poor people all over the world, and they ought to thank God for it. A good amalgam filling, thoroughly polished, will last a long time and well. The idea is, that there is no one material that has all the advantages; there is a place for almost everything.

Dr. Gordon White: I want to say, from all the information I have gotten on the subject of implantation, that climate influence plays a great part in the results of these operations. I don't approve of the operation very much. Some time ago, there was a gentleman who had at one time lived in San Francisco, and who told me of such an operation that had been performed very successfully in that climate, to his certain knowledge. Since this, the gentleman has come east to live; he had become convinced that the success of the operation was very greatly due to the western climate.

In most of the instances in the east, the operation has been a failure—certainly, in the majority. I throw this out for the information of some present.

Dr. G. F. S. Wright: If I heard Dr. Hunter's paper correctly, he seems to dwell at some length on the material which was run around a soft tooth. I think that point is well taken. Take that assertion in connection with what Dr. Crawford said about the increased fatality of disease, and the increased number of inefficient operations; I would only like to call attention to the fact, that forceps are not as important to dentists as other instruments have become. Forceps, formerly when a tooth was wrong, were called into action; the increased number of these things, which have come under the hand of the profession, have taxed the skill to a greater degree than formerly. Diseased conditions for which teeth were condemned and extracted, are now treated and filled; there are more dentists of a higher order of skill than formerly.

Would you be willing to abolish amalgam? Do you not often use amalgam as a restorer to partially broken down teeth to make your crown more stable? It is done by many, and done successfully. Badly decayed teeth that were filled frequently with amalgam are more frequently crowned. It is a better plan, but amalgam has its use as well as the crown.

Dr. H. E. Beach: Mr. President, I remember having heard an old minister of the gospel say on more than one occasion, in his prayer meetings, when some of the brothers were in the habit of trying to vary the prayers so as to take them out of the usual course of events, that prayers must of necessity be very much alike, in accordance with his idea of what prayer was, because we were always using the same things, and every time we got down to pray, we had to pray for the same thing; there was very little variation in it.

Now, if I understand, and I believe I do, what dentists are aiming to do in the world, it is to save teeth from destruction, and when we come up here and discuss questions of saving teeth by filling, we must of necessity say a great many things that are familiar to those that are successful in that operation; we must say these things over and over again, because there are some who do not quite understand what we mean by our description of different methods of filling teeth. I believe it is within the province of two metals, under the conditions that are necessary on the part of the patients, to preserve teeth; it is within the province of two metals to preserve almost every tooth in the human head. These two metals are tin and gold. The therapeutic effect of tin is a well-known fact to those who have used it to any considerable extent. What is the result? Again the advocacy for the use of tin, particularly with the soft character of teeth referred to, by the assayist, has been prominently before the Association of our State for twenty-one years to my knowledge. I know it was a long time before some could be induced to try it, because it is a base metal. We were sometimes told that tin exercised no therapeutic effect upon substances, but time has demonstrated that it does. In the cavities that are not subjected to the process of mastication, you will find in a large number of instances, recurrence of decay where they are filled with gold, and none where tin is used. There are operations that we are called upon to perform, where tin is the very best material that can possibly be used for the preservation of tooth substances. So much for tin. Dr. Freeman and myself have harped on this subject, until whenever it is mentioned, you can see every dentist smile and say, "there comes old tin again." In filling a carious tooth when it has been thoroughly dried, the cavity should be excavated as long as white specks appear immediately within the enamel border and carried to the extent, that it should have the appearance of perfectly sound walls before you attempt to make a filling. It is the thoroughness with which this is done, that works successful, and you may use any method you please. The combination of gold and tin as filling material for carious teeth is much talked of by some of our dentists; also by Dr. Miller. Why, bless your soul, gentlemen, one of the first experiments I ever saw was the combination of gold and tin, before Dr. Miller was born, I expect. Tin is one of the oldest tooth preservers, and I would be slow to advise the abandonment of tin. Crown and bridge-work has been both a success and a failure. If some of the operations that have been done in bridge-work could be examined in that particular line of operative dentistry, you would feel like you didn't know whether to call it bridge-work or botch-work, still it was said to have been done by good operators. I witnessed an operation where three teeth had been put in on a bridge. The bridge was a straight piece of gold about twenty-four gauge turned flat and fastened to the canine teeth and the crown was over the second molar, ordinary porcelain teeth were soldered on, and on the masticating surface was a slight tip of gold running from one end to the other, touching each tooth slightly; a gold bar was put across, but it didn't cut any figure as an aid to mastication. That was called bridge-work ! If that is the character of bridge-work to be done by dentists, good Lord deliver me from bridge-work. Dr. Stubblefied ridiculed, or rather called attention to the fact that he expected next to hear of the implantation of teeth to make crown and bridge-work, but from what our brother says, it has actually happened. I want to be delivered from that kind of a bridge. I believe that discretion should be taken into consideration; the necessity for the bridge and the amount of destruction that the natural teeth should be subjected to, in order to successfully make a good bridge. Where there is no covering of the coronal extremity of the teeth to prevent that band, which you attach to the teeth, from slipping in the direction of the gum, make an overlap. I made an operation like that some time ago, and it was satisfactory. Dr. White examined the work, and he thought it was a good piece. Where the crown of the tooth is perfect it is bad practice to cut off that part of the tooth to admit of a gold covering. It is not everybody that succeeds as well as the gentleman who so strongly advocates bridge-work, but I believe it is in the power of almost everyone of a mechanical turn, to arrive at that point, if they have the courage of that gentleman who preceded me. It requires courage to be successful in that sort of an operation.

So far as the use of amalgam is concerned, the time has come when it is necessary to put on large crowns in order to give your patient good masticating teeth, and instead of using amalgam we use gold crowns. Instead of using amalgam to build up waste places where the decay runs into the gums, in order to give a fit to your band, if you will use cement, you can leave that in there and it can do no harm. If you leave amalgam, it will sooner or later destroy the gold crown. I consider crown work of to-day the ne plus ultra of operative dentistry, The necessity for crown and bridge work arises from one or the other of two things; carelessness on the part of the patients, or insufficiency on the part of the operator. If a patient feels the necessity for the preservation of teeth, that every human being who has teeth should feel; and takes that care that is necessary, placing himself in the hands of a good and thorough operator, there would rarely be any necessity for crown and bridge-work.

Dr. Beadles: I haven't heard copper amalgam mentioned. I have been using some lately, and I have had some observations that are worth thinking about. I have been filling teeth with gold, from the cervical wall out. I was out of the United States last year for eight or nine months, and I was in some of the dental offices on the other side of the world. I examined some teeth. I put my instrument in several of them and tried to cut that amalgam out, but it was there just as hard as could be. It was as hard almost as flint; the tooth was black, but it was preserved and it was there. Since I have come back, I have been experimenting with copper amalgams, and so far successfully. In the cavities of the back teeth, where it is almost impossible to get gold filling, I excavate the cavity properly and fill with amalgam.

I sometimes put in a slight filling of amalgam next to the wall, finish with gold, and in that way get a gold filling so far as appearances are concerned, and I let the amalgam stay there.

I am a little enthusiastic on bridge work. I longed for the day when I could throw away the rubber plate. About a year ago, I took up bridge work and am convinced I will do no more plate work; I have got to that point. Dr. Beach, I think, said if you fill teeth with amalgam and put a crown over it, sooner or later the amalgam would destroy the gold. I would like to have his authority for it. I have filled with cement and crowned with gold, and I have filled with copper amalgam and filled with gold; I think it has powerful antiseptic qualities. I don't think we ought to condemn any method wholesale, any one method that may be successful in other men's hands.

Dr. Hunter: I remember very well some twenty years ago, when it would be quite a difficult matter to get a prominent operator to acknowledge that he would even build up a tooth with amalgam; he must have gold. He followed the radical idea that has been our tendency always; he ought to discriminate about materials. I admit the excellence of crown work; it has some evils about it, because it goes under the gum. Many times teeth are crowned in which amalgam would be the best to use. Discrimination is the thing.

Dr. Morgan: I wish to compliment the writer of the paper on "Operative Dentistry." I regard it as one of the most conservative papers on the subject; there has rarely been one in which there are fewer vulnerable points.

There are one or two points I would like to mention; the use of tin at the cervical border. In my practice I do not find the necessity of it. The gentleman who taught me to fill teeth, in his practice uses nothing but gold. It is not so much the chemical action of tin, but I don't believe tin has any therapeutic effect; it is the oxide that plays an important part. We don't take into consideration the changes that take place. These fillings of tin are frequently inserted in the mouths of children of tender ages, and when we examine these things 5, 10, 15 years afterward, we find a change in the condition of things.

As to bridge-work, I want to say again, we must be conservative. I don't believe we are over justified in the use of bridge-work for more than one tooth; I believe that is against all mechanical idea and is a failure. The gold crown is the best contribution to dentistry in the last 25 years.

Dr. Hunter: I agree with the gentleman as to the value of tin on the cervical margin and I agree with him as to the value of amalgam on the cervical margin. I do not think it is so much the therapeutic effect of tin or the oxide of tin that is so powerful, but some electro-magnetic effect. Make a tin covering and an amalgam covering on a cervical margin; polish them ever so nicely and they will remain bright, and where it is under gold it will turn perfectly black. If there is an oxidation of one of the metals, there must have been some chemical effect. If there is a chemical effect, there is a therapeutic effect. It is probable that the teeth may become harder under that kind of stimulation; in any case, I know that it is valuable. Call it what you please, it is proven to my satisfaction.

Dr. Richards: This is a kind of experience and love feast on amalgam. Copper amalgam has a position for itself already in two directions, if not more; two in particular I would like to allude to. I find by the use of copper amalgam, I am enabled to make a very soft filling, where the thickness of dentine over the nerve is so you couldn't permit the slightest pressure, where your cement produces an immense amount of pain, I take some amalgam so soft that it spreads out over the table; I put it in the tooth and let the patient go away; it will harden in the next twenty-four hours, with the therapeutic effect that all acknowledge it possesses. You can put copper amalgam in a cavity when the space is not sufficient for anything else; therefore, it surpasses anything else. I still adhere to the Bonwill crown. In regard to this, I have had the pleasure in the last two years of removing a couple of fillings of tin and gold combination, inserted by Dr. Miller, of Berlin. I grant you, the cervical wall was in a state of preservation; it had not been there long enough to have any deleterious effect. I didn't find that hardness I had heard of; I found the tin broken down and in a crumbling condition. I am like Blind Tom; I had rather see than hear of these combinations of tin and gold. In this little experience meeting there are points it is well to bring out, for the sake of dentists who do not practice as we do. Several years ago I began using tin on the cervical wall, with good success; I have had those fillings do remarkable service beyond anything else I had. I have put in fillings in the cervical wall, and the patient would fall into the hands of a dentist who was not familiar with that kind of practice, and some of the best fillings were taken out. Look well, brethren, before you leap!

Dr. Gordon White: I should like to say there is one little feature about tin that most of you perhaps are not familiar with. If you will take a little ingot of tin and an ingot of gold and try to mash them with pliers, you will be astonished to see how much easier tin will yield. It is the easy and perfect adaptation of tin that makes it so successful. Two or three years ago, I commenced the use of copper foil at the cervical wall. Last year I introduced it. It is a successful filling, but I do not like to work it as well as I do tin. I use a great deal of tin in those compound operations. I commenced the use of tin seven or eight years ago, and I have yet to see a single failure in the mouths of patients, for whom I have introduced that kind of an operation. Just here, while we are discussing crowns and bridge work, I have recently, or about two years ago, invented a crown. It is entirely original, and if not out of order, I will go to the board and illustrate it. I am one of the kind, if I happen to have a good thing, the profession can have it. This crown is what I call "a cast guiding surface crown." (Goes to the blackboard and draws a diagram of a tooth and explains the making of the new crown.) When I come to cut the band, where I wish to solder it, I cut it a little obliquely, and allow the band to be a little bit larger at the cutting end. After soldering the band, it is then placed upon the root and ground to articulate with the opposing tooth, bevelling the band toward the palatine side, allowing the buccal side to remain the length you desire the cusp. Then remove the band, and with the knuckling pliers crimp the point and make the knuckles as desired. I then replace the band upon the root, in the meantime you can see whether or not it is a good fit. Then place in the band Paris fluxed wax, directing the patient to close the mouth, which gives the articulating point, or the bite of the opposite tooth. With a syringe throw upon it a stream of ice water. Next take out the crown, and with Evans' wax spatula, trim the feathered edge of the wax next the root, and shape the cusps. With a small ball of wax placed on the palatine side of the inner cusp to form a funnel in the investment, the case is ready to be invested. This is done by taking equal parts of white sand and pumice stone with an equal part of plaster, being careful to place the buccal side of the band down. The wax is now burned out, leaving a coating of flux. I have my office-boy cut up a lot of twenty-carat solder very fine, rub it on the glass with borax and water and, with the blow pipe (without the flame), dry it off. This leaves a little coating of borax on each piece of solder. I have found this absolutely necessary to get a perfect fusion without occasionally burning the twenty-two-carat band. The mould formed by the wax is now filled with this solder. The blow-pipe is applied (be careful to heat *well* from the bottom), melting the solder, which flows into every part left vacant by the wax. This gives a perfect cast, which only needs to be polished and placed in position. The patient has only to close the mouth and it fits like a "clock wheel." I make molar and bicuspid crowns the same way. In cases where the root is very short and you need a pin, place the pin in position before inserting the band and wax. I make the combination gold and porcelain crown by the same process. Of all the crowns I have made, and I have made them by every plan, this gives me most satisfaction. It is certainly simpler than former processes, and overcomes the difficulty of getting a correct articulation, a feature in crown work that has given us all much annoyance.

Dr. J. Y. Crawford, of Nashville, Tenn.: It is in the manner of preparing roots of teeth for treatment that I want to make a contribution. I have been a dentist twenty-two years, and there has been a question in my mind whether I would practice the German method or the American. I have used the Morey drill, and I believe it is possible to get approximately at the roots of all teeth with that instrument, except those having a right angle curve, and they are very rare. We have been claiming to remove all the tissues of the nerve canals, but we have not been doing it. I took occasion once to investigate various forms of instruments for the preparations of root canals for treatment, and found the Morey drill to be the most effective. I said to the inventor of this drill, "I want you to test your instrument to its fullest extent." He commenced work, and in about the length of time I have been talking, he was down to the end of the canal; he moved over to the other side, and in the same length of time he was down to the end of that canal. With a well-selected and good set of Morey's instruments, it is not likely you will go through the tooth, unless you use great violence. I saw him prepare the canals of that dry, hard tooth, and he was from within one sixteenth to one-eighth of an inch of the end of that tooth in a little while. I ordered a full set of the Morey drills. The way to use them is to have a full set, and feel your way; never force the instrument, but let it feed itself; take it out and put in a smaller one, and keep on until you get an instrument that will feed itself. He assured me there was little danger of breaking them if you use a full set of drills.

Dr. Beach: I want to commend Dr. White's crown, because I know it is a most excellent crown; and as a method, it is a most excellent method. He is so accustomed to doing it himself he did not conceive the idea of a novice not filling the space quite full enough. Always put in enough solder to fill that space and have a little more. Fill it full enough not to have to drive any off. It is one of the best suggestions for making a crown.

The gentleman said he couldn't see what effect amalgam would have on a gold band. A demonstration is a proof. If he takes a band of gold he would soon find that the mercury would attack the gold so as to cause it to give way. You can't put a clasp on that tooth; it will absolutely unsolder. Mercury and copper will not amalgamate; it gets hard chemically, but it does not amalgamate. Mercury is free and you can put it over a flame and spew it out. If you put gold in contact with copper and mercury, the mercury will act upon the metal for which it has an affinity.

Dr. Wright: I move that we adjourn now and stand adjourned until after the clinics to-morrow, and give the use of this hall to the North Carolina Association this evening.

Dr. Turner: I would like to suggest the order of business for Thursday, if it is the pleasure of the house. I will read what I have to say. I suggest that we will meet Thursday morning from 9 to 1.30; afternoon session from 3.30 to 5.30. Friday morning from 9 to 1.30; afternoon session from 3.30 to 5.30. I move that we adopt this report. The motion seconded and carried.

Dr. Thompson: I move that we postpone the discussion of "Operative Dentistry" until a later date. Carried.

The Association adjourned at 5.30 p. m.

WEDNESDAY, August 12, 1891.

Morning and afternoon sessions were devoted only to clinics.

EVENING SESSION.

The meeting was called to order at 9 oclock p. m.

Dr. Wright in the chair.

Operative Dentistry passed. Prosthetic Dentistry called and postponed until a future time.

Dental Hygiene was next called, and passed for the present.

Dr. Wright: The next subject in the order of business is

PATHOLOGY AND THERAPEUTICS.

The discussion on this subject opened by a paper written by Dr. W. H. Gingrich, of Virginia, chairman, and read by Dr. Herring.

Dr. Beadles: It looks like a pity to waste time when a subject of so much importance is before us. I have been thinking a great deal along this line of thought lately. I have a case in my mind just now which I will relate in a few words. In my practice sometime ago, after being absent from the city for some little time, I came home, and a patient came to me for whom I had put in two or three very large amalgam fillings a year ago. As soon as I looked in his mouth I was very much surprised at it. I said: "Are you not in a very much better condition of health?" He said: 'I don't know. I haven't been sick." On this second examination I found the teeth looking well and all the amalgam fillings perfect. I could not understand it, and I related the circumstance afterward to a physician. He said: "He was in my employ at the time and he had contracted syphilis." Another case is of a young man with whom I have an acquaintance and for whom six months ago I operated on his teeth. They were good teeth and I expected my work to stand well. When he came back in a few months this tooth was in a soft condition and a bad condition all around. I couldn't understand why the tooth should change in structure. When I began to inquire, I found him in the same condition. In two or three months a tooth will change, and I agree wiih the paper, that there is nothing that has so powerful an effect on the teeth as the subject under discussion.

Dr. Richards: There is a question which has arisen in my mind as to whether the change he recognized in those teeth was the effect of disease or the effect of the treatment. From my observations and reading, I am led to conclude, that possibly the teeth would be affected by the constitutional changes which had taken place in overworked people in the season of rest. I am informed that the changes in the teeth are noticeable in people of that class. As regards the effect of that specific disease upon tooth substance, I have had one family under my observation in which the parents are noted for extraordinarily fine teeth. The husband was given to dissipation and the dissipation had told upon his children's teeth, which are the poorest excuses for teeth I have ever seen; even the bones are affected. There is but one conclusion that I can see; that is, the effects of this disease have been developed in these teeth. They break down in the shortest time and do not pay for the labor bestowed upon them. All these circumstances I have endeavored to combat. The mother's teeth do not suffer.

Dr. Lester: On this subject I have a case in mind that I would like to get some light on. The teeth of this person, a gentleman forty-five years and in good health, were very fine in quality and there were none missing. They became exceedingly painful and he had severe toothache. A tooth was extracted and a large part of the root was absorbed. Since that time another one had begun in the same way to ache. We have now two teeth out of the mouth with the roots absorbed. He has another one which he thinks from the sensation is going the same way. I would like to get some idea of the cause of the trouble and some information on the subject.

Dr. Wright: Dr. Lester, did those teeth simply show absorption or signs of necrosis? A. It was absorption, I should say. There was no evidence in my mind of necrosis. I have the teeth in my pocket. (Shows teeth to the president.)

Dr. Stubblefield: Without making any effort to reply to the gentleman, I will revert to the subject under discussion. Of course we must not confine it or limit it to those effects produced by that constitutional disease, syphilis. I suppose there is no one who is at all acquainted with the disease, that does not know that its manifestations are right along the line of dermoid tissues. The hair is known to be affected by it, lesions are produced on the skin by it, and it seems to me that the teeth in the development of the disease would be very much injured by its influence. The teeth are affected like any other portion of the structure. As to the cause of disease in the teeth, the latest investigations show it to be morbific agents localized in their effects until entrance is obtained. The decay usually presents itself, or frequently presents itself, between the teeth, at the points of contact between contiguous teeth. These points of contact between contiguous teeth represent surfaces that act by capillary attraction, by holding fluid. You may expel it by an effort, but that constitutes a little area for fluid, in which micro-organisms may live and work the different effects they may produce. It was impossible for years to produce decay out of the mouth as it was produced in the mouth, and this called the attention of experimenters to that fact. Until they found that disease germs developed an agent that was capable of breaking down this they couldn't arrive at a conclusion. There are certain changes that are followed by effete matter being thrown off, and if we have a field in which these organisms can live, at this point of contact between teeth holding fluid, we have a cause acting directly upon this initial point. In other portions of the teeth there is defective structure—deep sulci. We have accidents opening gateways to the inner structure, the more easily attacked parts of the teeth. The breaking down of inner structures is comparatively easy when once a gateway is obtained. There is no question but what this breaking down is more easily obtained by way of heritage acting upon organisms; therefore, where the vigor of parents has been impaired, we expect that the offspring would show defective organism. Causes that would break down perfect organs have an easy work when they attack points less capable of resistance.

Dr. Crawford moved that Dr. Patrick's paper on the "Morbific Process of Pus Formation" be read at this point to the Association. The motion was seconded and carried.—*Southern Dental Journal.*

